# Minocycline treatment inhibits microglial activation and alters spinal levels of endocannabinoids in a rat model of neuropathic pain

**DOI:** 10.1186/1744-8069-5-35

**Published:** 2009-07-01

**Authors:** Leonardo Guasti, Denise Richardson, Maulik Jhaveri, Khalil Eldeeb, David Barrett, Maurice R Elphick, Stephen PH Alexander, David Kendall, Gregory J Michael, Victoria Chapman

**Affiliations:** 1 School of Biological and Chemical Sciences, Queen Mary University of London, UK; 2 Neuroscience Centre, Institute of Cell and Molecular Science, Queen Mary University of London, UK; 3 School of Biomedical Sciences, University of Nottingham, UK; 4 School of Pharmacy, University of Nottingham, UK; 5 Pfizer, Ramsgate Road, Sandwich, Kent, UK

## Abstract

Activation of spinal microglia contributes to aberrant pain responses associated with neuropathic pain states. Endocannabinoids (ECs) are present in the spinal cord, and inhibit nociceptive processing; levels of ECs may be altered by microglia which modulate the turnover of endocannabinoids *in vitro*. Here, we investigate the effect of minocycline, an inhibitor of activated microglia, on levels of the endocannabinoids anandamide and 2-arachidonoylglycerol (2-AG), and the related compound N-palmitoylethanolamine (PEA), in neuropathic spinal cord. Selective spinal nerve ligation (SNL) in rats resulted in mechanical allodynia and the presence of activated microglia in the ipsilateral spinal cord. Chronic daily treatment with minocycline (30 mg/kg, ip for 14 days) significantly reduced the development of mechanical allodynia at days 5, 10 and 14 post-SNL surgery, compared to vehicle-treated SNL rats (*P* < 0.001). Minocycline treatment also significantly attenuated OX-42 immunoreactivity, a marker of activated microglia, in the ipsilateral (*P *< 0.001) and contralateral (*P* < 0.01) spinal cord of SNL rats, compared to vehicle controls. Minocycline treatment significantly (*P* < 0.01) decreased levels of 2-AG and significantly (*P* < 0.01) increased levels of PEA in the ipsilateral spinal cord of SNL rats, compared to the contralateral spinal cord. Thus, activation of microglia affects spinal levels of endocannabinoids and related compounds in neuropathic pain states.

## Introduction

Since the discovery of the cannabinoid receptors CB_1 _and CB_2_, and their endogenous ligands, there has been a rapid growth of evidence that the cannabinoid receptor system modulates the pathogenesis of various pain states. CB_1 _receptor agonists produce analgesic effects in models of neuropathic pain that are mediated by both peripheral and central sites of action [1]. However, activation of CB_1 _receptors in the central nervous system (CNS) is also associated with adverse psychoactivity [2].

Neuropathic pain responses are also attenuated by CB_2 _receptor agonists [3-5]. In contrast to the ubiquitous analgesic effects of CB_1 _receptor agonists, spinal administration of CB_2 _receptor agonists only has inhibitory effects in neuropathic rats and not sham-operated rats [6]. Importantly, the spinal effects of CB_2 _agonists in neuropathic mice are absent in CB_2 _knockout mice [4]. These functional data are supported by studies demonstrating an up-regulation of CB_2 _receptor mRNA and/or protein in the ipsilateral spinal cord [7-9] and ipsilateral dorsal horn [10] of neuropathic rats, compared to sham-operated rats. CB_2 _receptors in the spinal cord were co-localised with activated microglia in neuropathic rats [9], which is in keeping with earlier reports of CB_2 _receptor expression in microglia [11,12].

It is well established that spinal microglia undergo various stages of morphological and immunophenotypic change following nerve injury and contribute to the development of neuropathic pain states [13]. Indeed, single injection of activated microglia into the spinal cord produces thermal hyperalgesia in mice, one of the symptoms of neuropathic pain [14]. The tetracycline derivative minocycline is neuroprotective in models of traumatic brain injury [15], Parkinson's disease and Alzheimer's disease [16]; effects which are largely attributed to inhibition of microglia activation. Up-regulation of activated microglia in the spinal cord of neuropathic rats contributes to aberrant pain behaviour and plays an important role in central sensitization. Minocycline attenuates neuropathic pain behaviour and reduces microglia activation in the spinal cord [17-20]. *In vitro*, microglia are capable of synthesising and catabolising endocannabinoids [21,22], which play an important role in modulating nociceptive responses. Thus, we hypothesized that the presence of activated microglia in the spinal cord of neuropathic rats may contribute to the reported increased levels of the endocannabinoids anandamide (AEA) and 2-arachidonoylglycerol (2-AG) in the spinal cord [23]. Herein, we demonstrate that the inhibition of pain behaviour and microglia activation by chronic treatment with minocycline was associated with decreased spinal levels of 2-AG, but not AEA. By contrast, we report a significant elevation of PEA in the spinal cord of neuropathic rats treated with minocycline and demonstrate differing rates of hydrolysis of AEA and PEA by microglia BV-2 cells, which supports the notion that microglia differentially modulate levels of endocannabinoids in pathological states. Our data implicate a contribution of PEA, which has been shown to be neuroprotective in a model of focal cerebral ischemia [24], to the analgesic effects of minocycline in neuropathic rats.

## Results

### Minocycline attenuates mechanical allodynia induced by spinal nerve ligation

**Figure 1:**
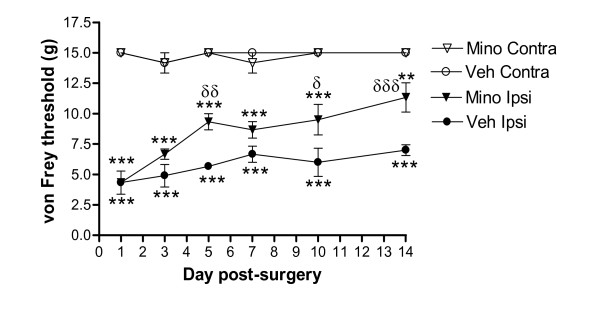
**Chronic treatment with minocycline significantly attenuates spinal nerve ligation-induced reduction in mechanical paw withdrawal threshold**. Minocycline (Mino, 30 mg/kg, ip) or vehicle (Veh, sterile water) was injected daily for 14 days and paw withdrawal threshold of the ipsilateral (Ipsi) and contralateral (Contra) hindpaw were measured using von Frey filaments (sham data not shown for clarity of figure). Data were analysed using 2-way ANOVA followed by Bonferroni's post-hoc tests, and are expressed as mean (± SEM, *n *= 6 rats/group). ** *P *< 0.01, *** *P *< 0.001 *vs *contralateral paw; ^δ ^*P *< 0.05, ^δδ ^*P *< 0.01, ^δδδ ^*P *< 0.001 *vs *vehicle-ipsilateral paw.

Chronic vehicle treatment did not alter the ipsilateral or contralateral paw withdrawal thresholds (PWTs) in sham-operated rats (data not shown). By contrast, SNL rats chronically treated with vehicle had a significantly (*P *< 0.001) lower ipsilateral PWT, compared to the PWT of the contralateral hindpaw of SNL rats and ipsilateral hindpaw of sham-operated rats (data not shown), consistent with the development of mechanical allodynia in these rats (Fig 1). Chronic daily treatment with minocycline (30 mg/kg, ip for 14 days) starting at 1 hr before SNL surgery significantly reduced the development of mechanical allodynia at days 5, 10 and 14 post-surgery compared to vehicle-treated SNL rats (Fig 1). Chronic minocycline treatment (30 mg/kg, ip) in sham-operated rats did not alter ipsilateral or contralateral PWT (data not shown).

### Effects of chronic minocycline treatment on spinal glial cell proliferation

**Figure 2:**
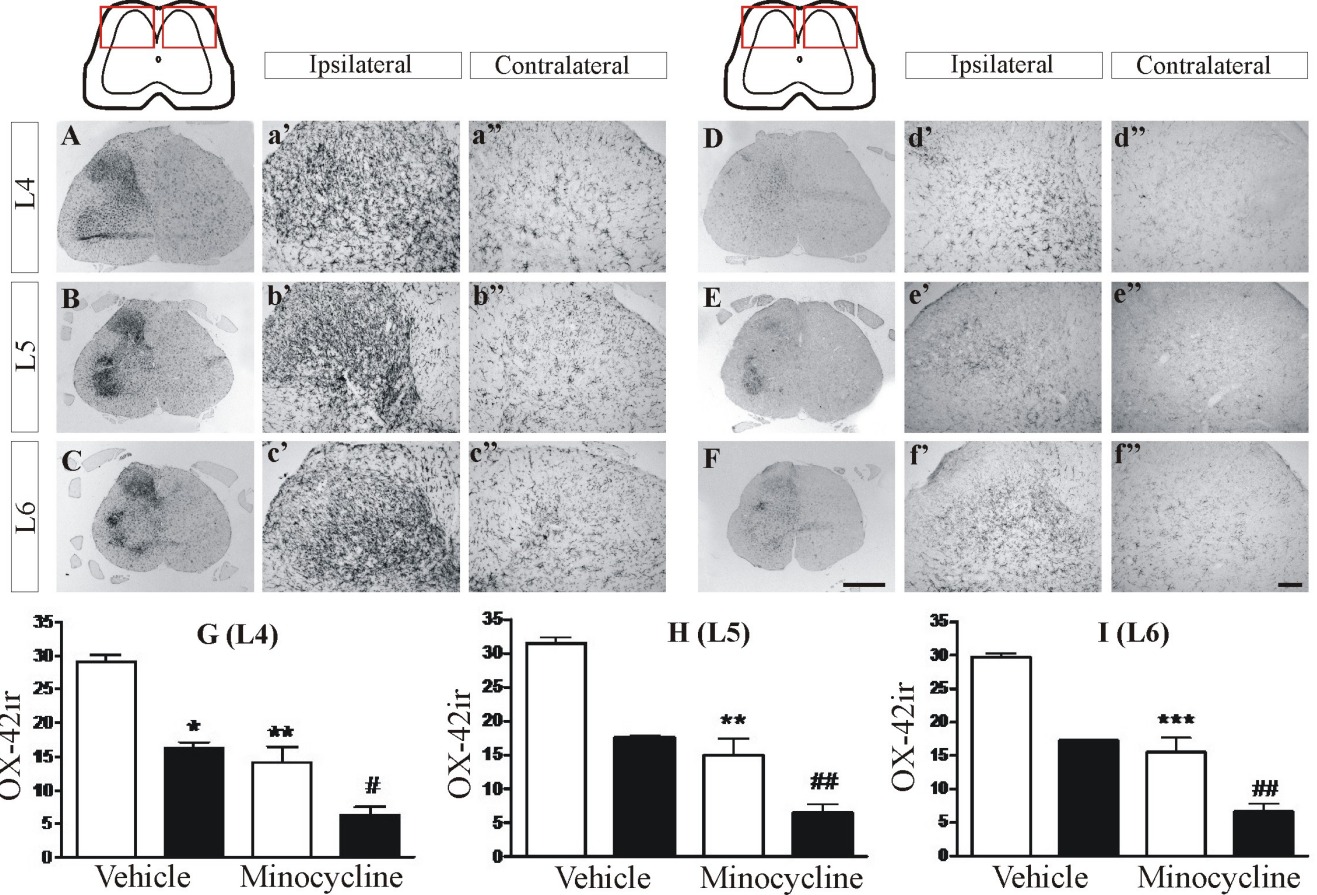
**Chronic minocycline treatment attenuates the spinal nerve ligation-induced increase in OX-42 immunoreactivity (ir) in the lumbar spinal cord**. Vehicle (A, B, C, sterile water) or minocycline (D, E, F, 30 mg/kg, ip) was injected daily for 14 days and spinal cords dissected and processed for immunohistochemical detection of OX-42ir at the level of L4 (A-a" and D-d"), L5 (B-b" and E-e") and L6 (C-c" and F-f"). High magnification images of the dorsal area used for OX-42ir signal quantification are also shown. Histograms (G-I) showing effects of minocycline or vehicle in L4-L6 spinal cord OX-42ir (white bars: ipsilateral side; black bars: contralateral side) are shown at the bottom. Data are expressed as mean ± SEM (*n *= 12 sections from 4 rats per group). Data were analysed using Kruskal Wallis non-parametric test followed by Dunn's multiple comparison posthoc analysis, * *P *< 0.05, ** *P *< 0.01, *** *P *< 0.001 *vs *ipsilateral-veh, # *P *< 0.05, ## *P *< 0.01 *vs *contralateral-vehicle. Scale bars: F = 1 mm (applies to A-F); f' = 100 μm (applies to a'-f' and a"-f").

SNL surgery significantly increased OX-42 immunoreactivity in the L4-L6 sections of ipsilateral spinal cord of chronic vehicle-treated rats, compared to the contralateral spinal cord, indicative of spinal microglia activation (Fig 2, OX-42 immunoreactivity, ipsilateral-vehicle, 30.2 ± 0.5; contralateral-vehicle, 17.0 ± 0.3). Chronic minocycline treatment (30 mg/kg, ip) significantly attenuated OX-42 immunoreactivity in the ipsilateral (*P *< 0.001) and contralateral (P < 0.01) spinal cord of SNL rats, compared to vehicle controls (Fig 2). Thus, effects of minocycline treatment on neuropathic pain behaviour were associated with decreased levels of a marker for activated microglia in the ipsilateral spinal cord of SNL rats. Nevertheless, levels of OX-42 in the ipsilateral spinal cord of SNL rats treated with minocycline were still elevated compared to the contralateral spinal cord of minocycline-treated SNL rats (total: ipsilateral-minocycline, 14.9 ± 1.3; contralateral-minocycline, 6.5 ± 0.7).

### Minocycline treatment alters spinal levels of endocannabinoids and related fatty acid ethanolamides in spinal nerve ligated rats

**Figure 3:**
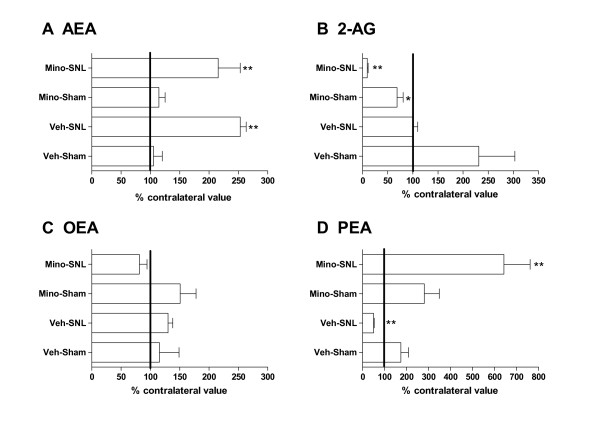
**Effects of chronic intraperitoneal dosing of minocycline (Mino, 30 mg/kg) or vehicle (Veh, sterile water) on the levels of endocannabinoids (A, B) and related fatty acid ethanolamides (C, D) in the ipsilateral spinal cord of spinal nerve ligated (SNL) or sham-operated rats**. Data were analysed using Mann-Whitney nonparametric test and are expressed as a mean percentage of contralateral levels ± SEM (*n *= 5 – 6 rats/group). * *P *< 0.05, ** *P *< 0.01 *vs *contralateral paw. AEA, anandamide; 2-AG, 2-arachidonoylglycerol; PEA, *N*-palmitoylethanolamine; OEA, *N*-oleoylethanolamine.

In SNL rats with chronic vehicle treatment, levels of AEA were significantly increased (*P *< 0.01) in the ipsilateral spinal cord, compared to the contralateral spinal cord (Table 1). Following chronic minocycline treatment in SNL rats, levels of AEA in the ipsilateral spinal cord were also elevated, compared to the contralateral spinal cord (Fig 3). The elevated ipsilateral levels of AEA in minocycline-treated rats were not different from the levels in vehicle-treated SNL rats.

**Table 1:** Levels of endocannabinoids and related fatty acid ethanolamides measured in the ipsilateral and contralateral spinal cord of spinal nerve ligated (SNL) rats treated chronically with vehicle (sterile water, ip, 2 ml/kg) daily for 14 days.

		AEA (pmol/g)	2-AG (nmol/g)	OEA (nmol/g)	PEA (nmol/g)
SNL – vehicle	Ipsilateral	65.86 ± 6.92**	52.21 ± 4.47	1.72 ± 0.15	4.20 ± 0.39**
	
	Contralateral	28.42 ± 0.86	54.40 ± 4.02	1.43 ± 0.08	8.80 ± 0.97

Data were analysed using Mann-Whitney non-parametric tests and are expressed as mean ± SEM mol/g (n = 5–6 rats per group). ** *P *< 0.01 vs contralateral spinal cord. AEA, anandamide; 2-AG, 2-arachidonoylglycerol; PEA, N-palmitoylethanolamine; OEA, N-oleoylethanolamine.

There were no significant differences in levels of OEA in the ipsilateral and contralateral spinal cord of vehicle-treated SNL rats (Table 1). Levels of OEA in ipsilateral and contralateral spinal cord of SNL rats were unaltered by minocycline treatment (Fig 3).

By contrast, chronic minocycline treatment of SNL rats had a marked impact on levels of 2-AG and PEA in the ipsilateral spinal cord, compared to the contralateral spinal cord (Fig. 3) and the ipsilateral spinal cord from vehicle-treated animals. In vehicle-treated SNL rats, levels of PEA were decreased (*P *< 0.01) in the ipsilateral spinal cord, compared to contralateral spinal cord (Table 1). However levels of PEA were increased (*P *< 0.01) in the ipsilateral spinal cord (31.59 ± 5.61 nmol/g) of minocycline-treated SNL rats compared to their contralateral spinal cord (5.01 ± 0.48 nmol/g) (Fig 3). Although there were no significant differences in levels of 2-AG in the ipsilateral and contralateral spinal cord of vehicle-treated SNL rats (Table 1), levels of 2-AG were significantly decreased (*P *< 0.01) in the ipsilateral spinal cord (4.53 ± 0.93 nmol/g) compared to contralateral spinal cord (47.66 ± 4.38 nmol/g) of minocycline treated SNL rats (Fig 3).

Chronic minocycline treatment of sham-operated rats did not significantly alter levels of AEA, OEA or PEA in the ipsilateral spinal cord, compared to the contralateral spinal cord (Fig 3). By contrast, levels of 2-AG in the ipsilateral spinal cord of minocycline treated sham-operated rats were significantly (*P *< 0.05) lower than levels in the contralateral spinal cord (Fig 3; ipsilateral: 39.79 ± 4.38 nmol/g, contralateral: 62.61 ± 7.91 nmol/g).

Levels of ECs and related compounds in the contralateral spinal cord of SNL rats treated with minocycline were compared to levels in the contralateral spinal cord of SNL rats treated with vehicle. Although levels of AEA in the contralateral spinal cord of minocycline treated SNL rats were not significantly different to levels in the contralateral spinal cord of vehicle-treated SNL rats, there was a trend towards an increase in the minocycline treated group (53.72 ± 2.33 pmol/g *vs *28.42 ± 0.86 pmol/g respectively). Levels of OEA in the contralateral spinal cord of minocycline-treated rats were comparable to levels in the contralateral spinal cord of vehicle-treated rats (1.51 ± 0.13 nmol/g *vs *1.43 ± 0.08 nmol/g). In contrast to the marked effects of minocycline treatment on levels of PEA and 2-AG in the ipsilateral spinal cord of SNL rats, this treatment did not significantly influence levels of PEA and 2AG in the contralateral spinal cord. There were no significant differences between levels of 2-AG or PEA in contralateral spinal cord of minocycline-treated SNL rats compared to vehicle-treated SNL rats (2-AG, 47.66 ± 4.38 nmol/g *vs *54.40 ± 4.02 nmol/g; PEA, 5.01 ± 0.48 nmol/g *vs *8.80 ± 0.97 nmol/g).

### Microglial differentially hydrolyse AEA and PEA

**Figure 4:**
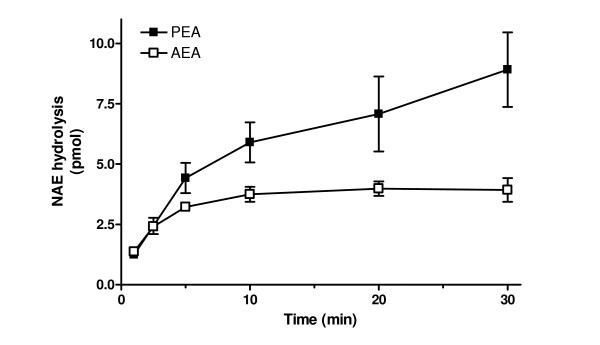
**Time course of hydrolysis of AEA (anandamide) and PEA (N-palmitoylethanolamine) in BV2 microglial cells**. Data are means ± SEM of six independent experiments conducted in triplicate.

Our *in vivo *data demonstrate that neuropathic pain has a differential effect on the levels of AEA and PEA in the spinal cord, which may reflect the presence of microglia in the spinal cord. Here, we investigated the hypothesis that microglia have a differential effect on the hydrolysis of AEA and PEA, which may account for our *in vivo *observations in neuropathic rats. We report marked differences in the time courses for PEA and AEA hydrolysis in intact BV-2 microglial cells (Fig 4.) Specifically, the capacity for hydrolysis of AEA by the BV-2 cells was limited, in that, after 10 minutes, there was no further metabolism of AEA, as shown by the plateau in accumulation of water-soluble product. In marked contrast, the hydrolysis of PEA continued for at least 30 minutes (Fig 4).

## Discussion

Two weeks following peripheral nerve injury, levels of the endocannabinoid AEA are markedly elevated in the ipsilateral spinal cord of neuropathic rats, whereas levels of the related anti-inflammatory compound PEA are significantly decreased. By contrast levels of 2-AG and OEA in the ipsilateral spinal cord of neuropathic rats are unaltered compared to the contralateral spinal cord. Inhibition of microglia activation, by chronic minocycline treatment, significantly altered pain behaviour and levels of 2-AG and PEA in the ipsilateral spinal cord of neuropathic rats without affecting levels of AEA or OEA.

Two weeks following peripheral nerve injury we observed a significant elevation in levels of activated microglia (increased OX-42 labelling) and AEA in the ipsilateral spinal cord, compared to the contralateral spinal cord. By contrast, levels of AEA in the ipsilateral spinal cord of sham operated rats were unaltered compared to the contralateral spinal cord. In the same group of neuropathic rats, levels of PEA were significantly decreased in the ipsilateral spinal cord, compared to the contralateral spinal cord. Levels of PEA were unaltered in the ipsilateral spinal cord of sham operated rats, compared to the contralateral spinal cord. The elevation in levels of AEA in neuropathic rats is consistent with an earlier report that levels of AEA are elevated 3 fold in the whole lumbar spinal cord at 3 and 7 days following chronic constriction injury of the sciatic nerve [23]. This previous study also reported decreased levels of PEA in the whole lumbar spinal cord at 3 days, but not 7 days, following chronic constriction injury of the sciatic nerve. Although our findings are consistent with the previous report of increased levels of AEA in the lumbar spinal cord, our data highlight the importance of selective ipsilateral changes in levels of ECs and related compounds which is not unexpected given the unilateral nature of the pain behaviour.

Our data demonstrating a differential effect of peripheral nerve injury on levels of AEA versus PEA in the ipsilateral spinal cord suggest that their metabolism can be regulated independently *in vivo*, in keeping with the discovery of different biosynthetic pathways for AEA and PEA in the brain [25] and evidence of independent signalling pathways in a microglia cell line (BV-2 cells) [26].

Despite the increased levels of activated microglia in the ipsilateral spinal cord, compared to the contralateral spinal cord, of neuropathic rats we did not observe any changes in levels of 2-AG in the ipsilateral spinal cord of neuropathic rats. *In vitro *studies have shown a role of 2-AG in CB_2 _receptor mediated migration of microglia [27] and proliferation of microglia [21]. In the present study we did not determine whether levels of 2-AG were elevated at earlier timepoints, which may have contributed to the recruitment of microglia to the site of spinal injury. This does, however, seem unlikely as levels of 2-AG were unaltered in the whole lumbar spinal cord at 3 days following peripheral nerve injury [23].

Chronic daily treatment with minocycline significantly attenuated the development of mechanical allodynia and the associated increase in activated microglia, in the L4 - L6 region of the ipsilateral spinal cord in neuropathic rats. These data are consistent with previous studies of the effects of minocycline on microglia activation and neuropathic pain behaviour [17,28]. Levels of OX-42 labelling in the contralateral spinal cord of vehicle-treated SNL rats were higher than those in the contralateral spinal cord of minocycline-treated SNL rats, consistent with the bilateral activation of microglia in the spinal cord following peripheral nerve injury [29] and indicates that minocycline treatment modulates this bilateral activation of microglia. Nevertheless, we did not observe any changes in paw withdrawal threshold of the contralateral hindpaw which indicates the absence of mechanical allodynia. Minocycline treatment did not significantly alter mechanical allodynia until day 5 post SNL surgery, there was however a trend towards an effect on day 3. These data corroborate previous studies reporting that the proliferation of microglia peaks at around 3 days following nerve injury, however this does vary slightly depending on the model studied [30]. The lack of effect of minocycline on mechanical allodynia on the first day after SNL surgery may reflect a delayed onset of action of minocycline [31]. Alternatively, microglia may not make a substantial contribution to the mechanical allodynia at this very early timepoint and, therefore, this treatment is unable to alter responses. It is feasible that the mechanical allodynia observed at day 1, and to some extent day 3, following SNL surgery is mediated, at least in part, by a transient post-surgical pain.

The effects of minocycline have been largely attributed to the inhibition of microglia [32], but effects on neuronal function *in vitro *should be borne in mind [31]. The integrated nature of *in vivo *studies makes it difficult to dissociate direct, versus indirect, effects of minocycline on microglia function and how this relates to levels of ECs and related compounds in neuropathic rats. It is estimated that, *in vitro *at least, microglia produce 20-fold more endocannabinoids than neurones or astrocytes [27,33] and primary microglia inactivate both AEA and 2-AG [34], suggesting that changes in the levels of activated microglia in the spinal cord, despite their lower abundance compared with neurones and other glia, could have a significant effect on whole tissue endocannabinoid content. Our data are consistent with this notion, indeed inhibition of microglia activation was associated with a significant decrease in levels of 2-AG in the ipsilateral spinal cord of neuropathic rats, compared to the contralateral spinal cord. These data suggest that activated microglia may contribute to the biosynthesis of 2-AG in neuropathic rats, which is consistent with previously described production of 2-AG by microglia *in vitro *[21,27,34], and provide indirect evidence for microglia modulation of 2-AG turnover *in vivo*.

We have identified major differences in the effects of microglial inhibition on levels of AEA and PEA in neuropathic rats, which suggest that there is divergence in the biosynthesis/catabolism of these N-acylethanolamines by microglia. Our data suggest that activated microglia play a major role in modulating levels of PEA, but not AEA or OEA, in the spinal cord of neuropathic rats. Given that *in vitro *studies using BV-2 cells suggest that microglia produce 1.5 fold more PEA than 2-AG [26], we hypothesised that the effects of inhibiting activated microglia on levels of PEA reflected a role of microglia in the hydrolysis of PEA.

To investigate whether differential catabolism of PEA and AEA by microglia may account for our *in vivo *data, we studied their rates of hydrolysis in intact BV-2 microglia cells. We demonstrate for the first time that the hydrolysis of PEA is sustained, whereas hydrolysis of AEA is transient, in BV-2 cells. These *in vitro *findings are consistent with our *in vivo *data, and suggest that the presence of activated microglia in the spinal cord of neuropathic rats contributes to the decreased spinal levels of PEA as a result of the sustained hydrolysis of PEA versus AEA. Conversely, inactivation of spinal microglia by minocycline would result in a decreased microglial component of PEA hydrolysis and the observed increase in levels of PEA in the spinal cord of neuropathic rats treated with minocycline. The differential hydrolysis of PEA and AEA by BV-2 cells suggests that distinct enzymatic activities may be responsible for AEA and PEA catabolism in microglia. Indeed, microglial cells express both fatty acid amide hydrolase and monoacylglycerol lipase, as well as other activities including a PEA hydrolase with a distinct pharmacological profile [22].

When considering the behavioural phenotype of minocycline-treated neuropathic rats versus vehicle-treated rats, the analgesia seen in the minocycline-treated rats may be mediated by the increased levels of AEA observed in neuropathic rats. It is unlikely that 2-AG contributes to the changes in behavioural allodynia, as the marked loss of 2-AG could be expected to exacerbate allodynia. The most prominent effect of minocycline was the significant elevation of ipsilateral PEA in minocycline-treated neuropathic rats, compared to vehicle treated neuropathic rats. PEA has been shown to attenuate neuropathic pain behaviour [35,36]. Although a role of cannabinoid CB_2 _receptors in the effects of PEA was initially proposed [37,38] this was then discounted [39]. Furthermore, the antinociceptive profile of PEA differs from the broad spectrum analgesia produced by systemically administered CB_2 _receptor agonists [40]. PEA has well described anti-inflammatory effects and has been shown to be neuroprotective [24]. PEA activates the nuclear receptor peroxisome proliferator-activated receptor-α, which mediates the analgesic effects of PEA [41]. Inhibitory effects of PEA in neuropathic rats are also mediated by PPARγ receptors [36]. Our data suggest that the contribution of PEA to the neuroprotective effects of minocycline warrants further investigation.

In conclusion, we have shown that the analgesic effects produced by minocycline treatment are associated with marked changes in levels of 2-AG and PEA and activated microglia in the spinal cord of neuropathic rats. Our data provide evidence for a role of activated microglia in the control of levels of endocannabinoids and related compounds *in vivo*.

## Methods

All experiments were carried out in accordance with the UK Home Office Animals (Scientific Procedures) Act (1986). Experiments were performed on 32 male Sprague Dawley rats (CRUK) in the light period of a 12 hr light/dark cycle. Animals had free access to food and water and were group-housed throughout the experiments. Rats were divided into 4 experimental groups comprising of sham-operated and SNL rats, treated with either vehicle or minocycline.

### Model of neuropathic pain: Spinal nerve ligation

The spinal nerve ligation model of neuropathic pain was used in this study. Spinal nerves L5-L6 were ligated according to the procedures described by [42]. Male Sprague Dawley rats (101 – 135 g) were anaesthetised using isoflurane (3% induction, 1–1.5% maintenance in 33% O_2_/67% N_2_O) and placed in a prone position. A midline incision was made at the L3-S2 level and the left paraspinal muscles at L4-S2 level were separated from spinal processes. Part of the L6 transverse process was removed with fine rongeurs and the L4-L6 nerves identified. The L5-L6 spinal nerves were isolated and tightly ligated (one suture per nerve) distal to the dorsal root ganglia and proximal to the sciatic nerve formation with 6-0 silk. The wound was closed in two layers using absorbable sutures and wound clips, following complete haemostasis. A similar procedure was performed for the sham surgery, except spinal nerves were not ligated. Post-surgery, the sham-operated and the SNL rats were group-housed (5 rats per cage) and their posture and behaviour were closely monitored for 48 hours.

### Drug treatment protocol

Effects of chronic intraperitoneal (ip) injections of minocycline (Sigma, UK, 30 mg/kg, *n *= 16) or vehicle (2 ml/kg sterile water, *n *= 16) on mechanical allodynia were measured over 14 days post-surgery in sham-operated and SNL rats. Minocycline (30 mg/kg, ip) or vehicle was injected into rats 1 hour before sham- or SNL surgery and subsequently daily for 14 days, approximately 15 hours prior to behavioural testing.

### Behavioural testing

From post-operative day 1 onwards (days 1, 3, 5, 7, 10 and 14), behavioural testing was performed to assess the development of mechanical allodynia, all testing was carried out between 8 and 11 am. Rats were placed in perspex cubicles with wire mesh grid floors and allowed to acclimatise prior to behavioural testing. Mechanical sensitivity of the ipsilateral and contralateral hind paw was assessed by measuring the paw withdrawal threshold (PWT) at which foot withdrawal to normally innocuous mechanical punctate stimuli was observed. Stimuli were delivered, from below, to the plantar surface of the foot using 0.2, 2, 4, 6, 8, 10 and 15 g von Frey hair stimuli for a maximum of 5 seconds per application. Each trial consisted of the application of a single von Frey hair, starting with a 4 g stimulus, and either increasing or decreasing in intensity until the PWT was observed, with the stimulus either side of the withdrawal weight subsequently repeated. The paw withdrawal threshold was recorded as the lowest von Frey stimulus to elicit a response.

### Measurement of endocannabinoids and related compounds

On post-operative day 15, approximately 15 hours after the last injection of minocycline (30 mg/kg, ip) or vehicle, rats were decapitated prior to a standardised and rapid dissection of the ipsilateral and contralateral lumbar (L4-L6) spinal cord. The whole process was complete within 3 minutes and samples were placed immediately on dry ice and then stored at -80°C to minimise post-mortem changes in levels of endocannabinoids (ECs) and related compounds. A validated lipid extraction method [43] was employed for measurement of AEA and 2-AG and related compounds, PEA and *N*-oleoylethanolamine (OEA). In brief, tissue was homogenised in an ethyl acetate/hexane mixture with internal standards (0.42 nmol d8-AEA, 1 nmol d8-2-AG), followed by repeated centrifugation and supernatant layer collection stages. Solid phase extraction was subsequently performed to purify samples. Simultaneous measurement of ECs and related compounds was then performed using liquid chromatography-tandem mass spectrometry (LC-MS/MS). Analysis was carried out on an Agilent 1100 system (Agilent Technologies, Waldbrunn, Germany) coupled to a triple quadrupole Quattro Ultima MS (Waters, Manchester, UK) recording in electrospray positive mode. Analytes were separated chromatographically on a HyPurity Advance C8 column and pre-column (100 × 2.1 mm internal diameter, 3 μm particle size; Thermo Fisher Scientific, Runcorn, UK) with a mobile phase flow rate of 0.3 ml/min. A gradient elution was used, with mobile phases consisting of A (water, 1 g/L ammonium acetate, 0.1% formic acid) and B (acetonitrile, 1 g/L ammonium acetate, 0.1% formic acid). Samples were injected from a cooled auto sampler maintained at 4°C. Multiple reaction monitoring of individual compounds, using specific precursor and product mass to charge (*m/z*) ratios allowed simultaneous measurement of AEA, 2-AG, PEA and OEA.

### Quantification of microglia

In a separate series of experiments, groups of vehicle-treated SNL rats or minocycline-treated SNL rats (n = 4 per group) were prepared for immunohistochemical studies of microglia activation. The development of mechanical allodynia in vehicle-treated SNL rats and the effect of minocycline treatment on mechanical allodynia were consistent with behavioural changes observed in vehicle-treated SNL and minocycline-treated SNL rats employed for measurement of levels of ECs. On post-operative day 15, rats were transcardially perfused with saline (0.9% w/v sodium chloride) followed by paraformaldehyde (PFA, Sigma, UK, 4% w/v) in phosphate-buffered saline (PBS, 3.2 mM Na_2_HPO_4_, 0.5 mM KH_2_PO_4_, 1.3 mM KCl, 135 mM NaCl, pH 7.4) under terminal anaesthesia induced by pentobarbital sodium (Euthetal^®^, Merial Animal Health Ltd, UK, 800 mg/kg, ip). The spinal cord was dissected and stored in PFA for approximately 2 hrs at 4°C, and then transferred to a cryoprotectant solution (30% w/v sucrose, 0.1% w/v sodium azide in PBS).

Spinal cords were frozen in O.C.T. embedding medium (VWR, UK) and 14 μm serial sections were cut onto Superfrost Plus slides (VWR) using a cryostat (CM3050S, Leica Microsystems, UK Ltd). Sections were air dried and then treated with 3% hydrogen peroxide in PBS for 30 minutes at room temperature, washed with PBS and then blocked with 5% normal goat serum (Sigma, UK) in PBS/0.2% Triton X-100. They were then incubated overnight with mouse monoclonal anti-OX-42 (AbD Serotec, UK), diluted 1:1000 in PBS/0.2% Triton X-100. This antibody recognizes the complement receptor type 3(CR3) and specifically labels the plasma membrane of the microglial cells. Sections were washed in PBS and incubated with a biotinylated goat anti-mouse IgG secondary (Vector Laboratories, UK) diluted 1:500 in PBS/0.2% Triton X-100 for 2 hours at RT. They were washed again, treated with avidin-biotin complex (Vector Laboratories, UK) according to the manufacturer's instructions, rinsed again and incubated with diaminobenzidine/nickel (Vector Laboratories, UK). The reaction was stopped with PBS and slides were dehydrated and coverslipped. Sections obtained from the eight rats were processed at the same time, and the experiments were repeated three times. Images were acquired using a Leica DMRA2 microscope (Leica, Nussloch, Germany), and digital images were captured using a Retiga 1300 12-bit camera (QImaging, Burnaby, BC, Canada) and QCapture 1.1.6 software (QImaging) running on a Macintosh G4 computer. Images were then assembled into figures using Photoshop 7.0 (Adobe Systems, San Jose, CA). OX-42 optical density was analysed at the level of L4, L5 and L6. To quantify, we applied Image J Software based analysis: nine equivalent boxes (areas of interest, measuring 180 × 180 squared μm) were placed within each dorsal horn covering the medial to lateral extent of Rexed laminae I-V, and the mean grey saturation measured within each area of interest. The mean value of three equivalent boxes outside each section was then subtracted to normalize background. Three sections were analysed at each level. Results (mean ± SEM) are expressed as arbitrary units.

### AEA and PEA hydrolysis in microglial cells *in vitro*

The AEA/PEA hydrolysis assay was based on Paylor et al. [44]. In brief, BV2 mouse microglial cells (a generous gift from N. Stella, University of Washington), passage number <8, grown to confluency in 24 well plates were pre-incubated for 10 min at 37°C in 400 μl HEPES-containing physiological buffer, pH 8.0, containing 0.1% fatty acid-free bovine serum albumin. The reaction was initiated by the addition of 250 nM AEA or PEA containing [^3^H]-AEA or [^3^H]-PEA (approx 60,000 dpm/well), respectively. After varying periods of incubation (2 to 30 minutes) at 37°C, the buffer was rapidly removed by aspiration and replaced with 400 μl ice-cold methanol. Wells were scraped and the entire contents of each well collected. Following additions of 400 μl chloroform and 200 μl H_2_O, the phases were separated by centrifugation at 5,000 g for 2 minutes. Radioactivity in an aliquot of the aqueous, upper phase containing [^3^H]-ethanolamine was quantified by liquid scintillation spectroscopy.

### Data analysis

Behavioural data were statistically analysed using two-way ANOVA with treatment and time as factors, followed by Bonferroni's post-hoc test, and are presented as mean ± SEM of mechanical PWT in grams. Levels of ECs and related compounds were calculated as the ratio of the EC peak area with its internal standard on the chromatogram and are expressed as mol/g wet weight of tissue. Levels of ECs and related compounds were excluded from analysis if the value was greater than 2 standard deviations from the mean, giving *n *= 6 for all groups except: *n *= 5 for contralateral vehicle-saline and ipsilateral vehicle-sham. Ipsilateral and contralateral spinal cord levels of AEA, 2-AG, OEA and PEA were statistically compared using a Mann-Whitney test and are presented as mean % of contralateral spinal cord values, or raw mean ± SEM). For comparison of levels of AEA, 2-AG, OEA and PEA between treatment groups, data were statistically compared using Kruskal-Wallis test. Immunohistochemical data were analysed using the Kruskal-Wallis non-parametric test and are presented as mean ± SEM of density of OX-42 staining. Hydrolysis of AEA & PEA was expressed as pmoles/well and was analysed as means ± SEM from six different cell passages investigated in triplicate.

## Abbreviations

AEA: anandamide; 2-AG: 2-arachidonoylglycerol; CB_1_: cannabinoid 1 receptor; CB_2_: cannabinoid 2 receptor; CNS: central nervous system; EC: endocannabinoid; MAPK: mitogen activated protein kinase; OEA: *N*-oleoylethanolamine; PEA: *N*-palmitoylethanolamine; PFA: paraformaldehyde; PWT: paw withdrawal threshold; SNL: spinal nerve ligation.

## Competing interests

The authors declare that they have no competing interests.

## Authors' contributions

LG carried out the quantification of microglia and statistical analysis. DR carried out the drug dosing, behavioural testing, quantification of endocannabinoids and related compounds and statistical analysis. MJ generated neuropathic rats and carried out behavioural testing. KE carried out hydrolysis assays in BV2 cells and statistical analysis. DAB participated in study design. MRE participated in study design. SPHA participated in study design. DAK participated in study design. GJM participated in study design. VC generated neuropathic rats, conceived the study, and participated in its design and coordination. All authors read and approved the final manuscript.
